# Assessment of a Commercial Real-Time PCR Assay (Vitassay qPCR Malaria 5 Test) to Detect Human Malaria Infection in Travelers Returning to France

**DOI:** 10.3390/diagnostics12112747

**Published:** 2022-11-10

**Authors:** Marylin Madamet, Rémy Amalvict, Nicolas Benoit, French National Reference Centre for Imported Malaria Study Group, Bruno Pradines

**Affiliations:** 1 Unité Parasitologie et Entomologie, Département de Microbiologie et Maladies Infectieuses, Institut de Recherche Biomédicale des Armées, 13005 Marseille, France; 2 Aix Marseille Univ, IRD, AP-HM, SSA, VITROME, 13005 Marseille, France; 3 IHU Méditerranée Infection, 13005 Marseille, France; 4 Centre National de Référence du Paludisme, 13005 Marseille, France

**Keywords:** malaria, *Plasmodium*, molecular diagnosis, real-time PCR, imported malaria

## Abstract

Malaria is the most common human parasitic disease in the world with the highest morbidity and mortality. Due to the severity of malaria caused by *Plasmodium falciparum* and the urgency of therapeutic management, quick and reliable diagnosis is required for early detection. Blood smear microscopy remains the gold standard for malaria diagnosis. Molecular diagnosis techniques are the most sensitive and specific in cases of low parasitaemia and in the detection of mixed infections. The purpose of this study was to evaluate a new commercial test involving the molecular diagnostic technique to detect the five human *Plasmodium* species. The Vitassay qPCR Malaria 5 test is based on the multiplex real-time PCR of a conserved target region of the 18S rRNA gene for the five human *Plasmodium* species. A total of 190 samples collected from imported cases of malaria were diagnosed using this test and compared against a homemade reference real-time PCR. The sensitivities of the Vitassay qPCR Malaria 5 test for all *Plasmodium* species ranged from 93.8% to 100% and specificity ranged from 97.7% to 100%. Based on these criteria, this test is recommended for the diagnosis of the human *Plasmodium* species.

## 1. Introduction

Malaria is one of the most severe public health problems worldwide, causing an estimated 241 million clinical cases and leading to 627,000 deaths in 2020 [1]. According to the World Health Organization (WHO), the African continent is the most impacted by malaria infection, accounting for 95% of deaths worldwide, mostly in young children.

France is the non-endemic European country most affected by imported cases of malaria. The French National Reference Centre for Malaria (Malaria CNR) is charged with the epidemiologic surveillance of imported malaria cases in France [2]. Some 2185 imported malaria cases were reported by the network of biological and clinical correspondents to the French Malaria CNR in 2021, and it has been estimated that there are 4995 cases for the whole of France (personal data, French Malaria CNR). The most commonly detected species in 2021 was *Plasmodium falciparum* (88.5%). It is imperative that the Malaria CNR has the most efficient diagnostic tools. Effective diagnostic techniques could enable the early diagnosis of malaria, and prevent infection from progressing to severe malaria or death. Diagnosis should be performed within four hours of the sample’s arrival in metropolitan France [3]. As recommended by the WHO, the gold standard for malaria diagnosis is based on the microscopic observation of asexual stages of the parasite (thin blood films and thick blood smears), which is the only method recommended in France, and rapid diagnostic tests (RDTs) for malaria, based on immunochromatographic techniques, are routinely used for diagnosis in the Malaria CNR. However, in cases of low parasitaemia and mixed infections, microscopic diagnosis is sometimes difficult. RDTs also lack the ability to detect low parasitaemia and species other than *P. falciparum* [4,5]. Moreover, many false-negative results have been reported with *P. falciparum* histidine-rich protein 2 (PfHRP2)-based RDTs, due to parasites lacking the *pfhrp2* gene [6]. Over the last three decades, molecular biology has been developed as a highly sensitive and specific complementary diagnostic method [3]. A homemade real-time PCR is commonly used by the Malaria CNR in Marseille [7]. Currently, very few different qPCR malaria tests are commercially available. Most of them are designed to identify only the Plasmodium genus or the four human Plasmodium species (*P. falciparum*, *P. vivax*, *P. malariae,* and *P. ovale*). It is essential to select the most efficient kit depending on test performance (sensitivity and specificity) and operating constraints (storage conditions and ease of use).

The aim of this study was to evaluate the sensitivity and specificity of the Vitassay qPCR Malaria 5 test, based on the real-time PCR of the 18S rRNA plasmodial gene. A thin blood smear, the gold-standard method, and a homemade real-time PCR were used as references.

## 2. Materials and Methods

### 2.1. Collection of Plasmodium Isolates

The samples were taken from malaria cases imported from endemic countries in patients hospitalised in French civilian or military hospitals and sent to the Malaria CNR (Institut de Recherche Biomédicale des Armées, IHU Méditerranée Infection, Marseille).

Ninety samples isolated between 2019 and 2020 were assessed in this study (Table 1) and retrospectively selected for characteristics such as low parasitaemia, species other than *P. falciparum,* and mixed infections, as well as 100 complementary isolates collected between 2021 and 2022 without selection.

### 2.2. Microscopic Technique

*Plasmodium* infection samples were identified from thin blood smears stained with eosin and methylene blue using a RAL^®^ kit (RAL Reagents, Paris, France) by well-trained and experienced technicians. The parasitaemia percentage was estimated by counting the number of infected cells as a percentage of the red blood cells.

### 2.3. Reference Real-Time PCR

The reference molecular diagnosis was performed by real-time PCR using a Light Cycler 2.0 (Roche Group, Rotkreuz, Switzerland) to identify four human *Plasmodium* species (with the exception of *P. knowlewsi*), as previously described [7]. The primers and probes used were presented in Table 2. Briefly, DNA was extracted from 200 µL of whole blood using the QIAamp^®^ DNA Blood Mini kit, according to the manufacturer’s recommendations (Qiagen, Hilden, Germany). Each parasite species was detected by an individual real-time PCR targeting a specific gene for each of the four human Plasmodium species using the Light Cycler^®^ TaqMan^®^ Master Mix (Roche Group, Switzerland). For each PCR run, two negative controls (ultra-pure water and human DNA) and one positive control (specific DNA from each species) were used.

Homemade real-time PCR tests, targeting the 18S rRNA gene, were performed to differentiate *P. ovale wallikeri* from *P. ovale curtisi* using Plasmo1_F (5′ GTTAAGGGAGTGAAGACGATCAGA 3′) and Plasmo2_R (5′ AACCCAAAGACTTTGATTTCTCATAA 3′) primers, as previously described [8].

### 2.4. Vitassay qPCR Malaria 5 Test

The Vitassay qPCR Malaria 5 test is a ready-to-use multiplex PCR that can be used with the CFX96™ Real-Time PCR Detection System (Bio-Rad, Marnes-la-Coquette, France), according to the manufacturer’s recommendations (Servibio, Courtaboeuf, France). DNA was extracted from 200 µL of whole blood using an extraction control (EC). This test contains, in each well, all the necessary real-time PCR reagents to amplify a conserved target region of the 18S rRNA gene for the five human *Plasmodium* species. The first low-profile strip contains the multiplex reaction mix for the detection of *P. malariae*, *P. knowlesi,* and/or *P. ovale*, while the second contains the mix for the detection of *P. falciparum* and/or *P. vivax*. Each plasmodial species was simultaneously detected by distinct fluorescence channels: *P. malariae* and *P. falciparum* DNA targets in the FAM channel, *P. knowlesi* and *P. vivax* DNA targets in the ROX channel, and *P. ovale* DNA targets in the Cy5 channel, and the EC DNA target is amplified and detected in the HEX channel. For each PCR run, the EC, a negative control (human DNA), and a positive control (specific DNA from each species) were included. The EC allows the detection of a problem of extraction of a poor-quality and/or a shoddy reaction due to inhibitor presence.

### 2.5. Analysis of the Results

The aim of analysing the results was to compare the ability of the Vitassay qPCR Malaria 5 kit to detect each *Plasmodium* species infection against the reference homemade real-time PCR results. Diagnostic performances such as sensitivity and specificity were evaluated using the following formula (TP = the number of true positives, FN = false negatives, FP = false positives, and TN = true negatives):

Sensitivity = TP/(TP + FN), (proportion of samples for which the malaria species was correctly identified)

Specificity = TN/(FP + TN), (proportion of samples for which the malaria species was not correctly identified)

For statistical analysis, the Fisher Student’s *t* test was used appropriately, *p*-values were evaluated, and statistical significance was set at *p*-values < 0.05.

## 3. Results

All the samples were detected by thin blood smears and confirmed by real-time PCR.

A total of 190 samples were assessed, and 187 isolates were found to be positive by microscopy with parasitaemia ranging from <0.001% to 33%. A total of 147 isolates were identified as *P. falciparum* (parasitaemia between <0.001% and 33%) by homemade real-time PCR, 5 as *P. vivax* (parasitaemia between <0.001% and 0.6%), 11 as *P. malariae* (parasitemia between <0.001% and 0.2%), 16 as *P. ovale* (parasitemia between <0.001% and 0.15%), 8 as mixed infections (4 as *P. falciparum/P. malaria*, 3 as *P. falciparum/P. ovale,* and 1 as *P. falciparum/P. malaria/P. ovale*), and 3 as negative samples (Table 1). Among the sample identified as *P. ovale*, 13 were *P. ovale curtisi* and 7 were *P. ovale wallikeri*.

Between the commercial molecular diagnostic test (Vitassay qPCR Malaria 5) and the homemade reference real-time PCR, only three discrepant diagnoses were observed. One case was due to *P. falciparum* and the two others were mixed infections (Table 3).

The 187 other diagnoses showed no significant difference in terms of the specific detection of the four human *Plasmodium* species (*P. falciparum*, *P. vivax*, *P. malariae,* and *P. ovale*). Samples with low parasitaemia (n = 28/190, <0.001%) were correctly detected. Overall, mixed infections with *P. falciparum* and non-falciparum species were distinctly diagnosed. The two species of *P. ovale*, *wallikeri*, and *curtisi* were successfully identified.

Using the Vitassay qPCR Malaria 5 test, the sensitivities for each respective malaria species were 99.4% for *P. falciparum*, 100% for *P. vivax*, 100% for *P. ovale*, and 93.8% for *P. malariae,* while the respective specificities were 97.7% for *P. falciparum*, 100% for *P. vivax*, 99.4% for *P. ovale,* and 100% for *P. malariae* (Table 4).

**Figure 1:**
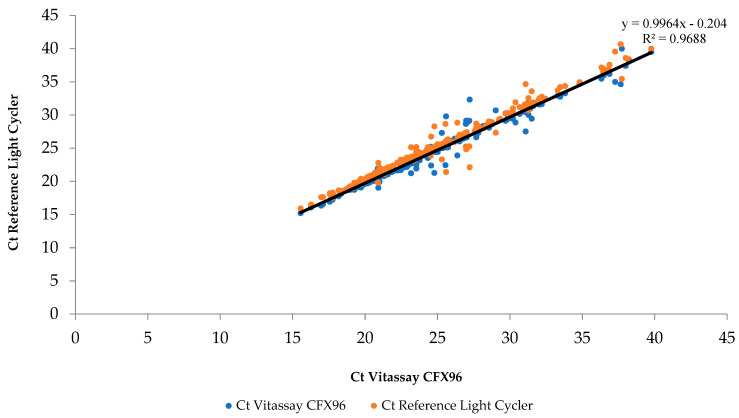
Comparative of the Ct values of the Vitassay CFX96^TM^ versus the reference Light Cycler.

The variability of the Ct values was normal between the two PCR methods, due to the intrinsic variability of the molecular diagnosis (thermal cycler and reaction mixture) and the specific target detected. However, the Ct value results between the two real-time PCR techniques were concordant with Pearson’s correlation coefficient (r) of 0.984 and a *p*-value of 9.8 × 10^−91^) (Figure 1).

The Ct values of EC included in each reaction were constant, which made it possible to validate the performance and reproducibility of the Vitassay qPCR Malaria 5 test.

The diagnostic performance between the Vitassay qPCR Malaria 5 test and the reference homemade real-time PCR was compared in 190 clinical samples using a Fisher’s exact test. There was no significant difference between the two assays (*p*-value = 1).

These results show the high sensitivity and specificity for detecting *Plasmodium* spp. using the Vitassay qPCR Malaria 5 molecular diagnostic test.

## 4. Discussion

The severity of malaria due to *P. falciparum* requires early therapeutic management involving rapid and effective diagnosis. Although diagnosis by microscopy remains the gold standard for malaria identification, molecular techniques can detect low parasitaemia and mixed infections and consequently avoid misdiagnosis. A new commercial molecular diagnostic kit was therefore evaluated: the Vitassay qPCR Malaria 5 test.

The Vitassay qPCR Malaria 5 test makes it possible to detect and differentiate *P. falciparum*, *P. vivax*, *P. ovale*, *P. malariae,* and/or *P. knowlesi* using multiplex real-time PCR on clinical samples. According to the supplier, this molecular diagnostic tool has a detection limit of ≥10 copies of DNA per reaction. We evaluated the limit of detection (LOD) of the Vitassay qPCR Malaria 5 test for *P. falciparum* by multiple dilutions of an isolate. This LOD was estimated at a parasitaemia value equivalent to 0.000001%, while the LOD of the microscopy was estimated at 0.001% (>20 min of examination and around 100,000 erythrocytes observed). The analytical specificity for malaria pathogens was tested within a panel of different microorganisms, and no cross-reactivity was observed between any of these species. In addition, the test was highly specific for all the individual *Plasmodium* spp.

To confirm the appropriate performance of the molecular diagnostic technique, an EC was included in each reaction. The emission of the EC signal makes it possible to confirm the validity of the extraction procedure and the absence of any nonspecific amplification and/or contamination.

Moreover, the freeze-dried reagents and the test can be shipped and stored at temperatures between 2 °C and 40 °C until the expiration date indicated on the label, a feature which is essential under restrictive conditions.

For early management and treatment, the reliable diagnosis of malaria remains a vital means of specifically detecting the presence of malarial parasites in cases of fever in endemic malarial regions. The time required for a complete molecular diagnosis according to the two techniques is less than three hours. Moreover, the Vitassay qPCR Malaria 5 test is performed with a lower risk of contamination in comparison with the homemade reference real-time PCR.

This kit was efficient in detecting malaria parasites with sensitivities ranging from 93.75% to 100% and specificities ranging from 97.7% to 100%. In malaria samples where parasitaemia was very low (<0.001%), the molecular diagnosis remained reliable.

There was no significant difference in the detection of mixed malaria infections. The major advantage of using this assay would be to detect mixed infections, as is the case in French Guiana and the Republic of Djibouti [9,10,11]. Microscopy can miss co-infections, especially when one species is more predominant in the isolate [12,13]. Detecting mixed infections has clinical importance. Infection with *P. falciparum* is known to lead to severe malaria. By contrast, *P. ovale*, *P. vivax,* or *P. malariae* cause less severe infections. Cases of *P. vivax* severe infections have been reported at low prevalence in the last decade. Adequate diagnosis for appropriate treatment (artemisinin-based combination therapy followed by primaquine) leads majorly to prevent relapse in cases of co-infection of *P. falciparum* with *P. vivax* or *P. ovale* [14,15]. Uncomplicated non-falciparum malaria can be treated by chloroquine but falciparum malaria cannot, due to resistance [16]. The risk of clinical treatment failure with mixed infection after initial treatment is higher compared with the treatment of *P. falciparum* [17]. In conclusion, misdiagnosis of mixed infections can be a cause of treatment failure. Moreover, patients with mixed infections had a higher proportion of multiple organ failure than those with *P. falciparum* mono-infection [18].

In the context of *P. ovale* parasites, there was no difference in diagnosis between the two species, *P. ovale curtisi* and *P. ovale wallikeri*. The majority of imported malaria cases in metropolitan France diagnosed by the Malaria CNR come from Africa, where *P. falciparum* is predominant, but also from specific African countries (Côte d’Ivoire, Gabon, and Senegal) where the transmission of *P. ovale* has been reported [19,20,21]. Between 2000 and 2015, 6468 cases of malaria were reported in the French armed forces, with 60.7% being *P. falciparum*, 29.0% *P. vivax*, 7.2% *P. ovale,* and 1.8% *P. malariae* [22]. Moreover, every year the French armed forces conduct military operations in endemic malaria areas including French Guiana, where *P. vivax* is predominant [9]. Consequently, the use of a specific PCR kit is important to detect all human Plasmodium species.

The limitations of this study include the low number of samples. Although 190 malaria samples were tested, given the African origin of these samples, none were identified as *P. knowlewsi*.

Diagnostic tools for malaria have expanded beyond blood smear microscopy, previously the gold-standard technique. Molecular and innovative techniques have emerged, including RDTs and methods derived from PCR such as nested PCR, real-time PCR, and loop-mediated isothermal amplification (LAMP) [23]. These molecular diagnostic methods have higher sensitivity and specificity and allow the detection of low parasitaemia. The early and safe diagnosis of malaria is of paramount importance to ensure appropriate treatment and reduce the incidence of severe forms of the disease and deaths. The concomitant use of a molecular method and the gold standard for the identification of malaria parasites would ensure reliable diagnosis [24]. Microscopy always had limitations, i.e., low parasitaemia, slide quality, and staining problems. Moreover, microscopic techniques require trained personnel and are time-consuming. Molecular diagnostic techniques have higher sensitivity and allow identify mixed infections. However, in resource-poor settings in endemic countries, the use of these techniques is less feasible. With advancements and affordable technology with portable machines, these techniques could reach more people.

## 5. Conclusions

The Vitassay qPCR Malaria 5 test has been shown to be a reliable tool for the diagnosis of malaria in non-endemic countries, detecting low parasitaemia and mixed infections with a sensitivity similar to the homemade reference real-time PCR methods, but is more practical to use due to its multiplex nature.

## Figures and Tables

**Table 1:** Identification of *Plasmodium* species of the 190 samples by homemade real-time PCR.

	*P. falciparum*	*P. vivax*	*P. ovale*	*P. malariae*	*P. falciparum* *P. ovale*	*P. falciparum* *P. malariae*	*P. falciparum* *P. ovale* *P. malariae*	Negative	Total
Selected samples	62	3	9	9	3	2	0	2	90
Other samples	85	2	7	2	0	2	1	1	100
Total	147	5	16	11	3	4	1	3	190

**Table 2:** Sequences of primers and probes for reference RT-PCR.

Plasmodium Species	Access Number Genes	Probe or Primer Name	Primer Sequence
*P. falciparum*	*Aquaglyceroporine* *(AJ413249)*	Probe Pf	FAM-5′ TACACTACCAACACATGGGGCTCAAGAGGT 3′- BHQ1
		*P falciparum* F	5′ TTTATGTATTGGTATAACATTCGG 3′
		*P falciparum* R	5′ GGCAAATAACTTTATCATAGAATTGAC 3′
*P. vivax*	*Enoyl-acyl carrier protein reductase (AY423071)*	Probe Pv	HEX-5′ CATCTACGTGGACAACGGGCTCAACA 3′-BHQ1
		*P vivax* F	5′ GTGGCCGCCTTTTTGCT 3′
		*P vivax* R	5′CCTCCCTGAAACAAGTCATCG 3′
*P. ovale*	*Circumsporozoïte* *(S69014)*	Probe Po	HEX- 5′ ATTTTTTGCATCAACCTTTCTTCTAGCCC 3′-BHQ1
		*P ovale* F	5′ GGAGGAATGGTCACCATGTAGTGT 3′
		*P ovale* R	5′ CAAATTTCAGTTTCAAGGTCACTTAA 3′
*P. malariae*	*Ookinete surface protein 25 (AB074976)*	Probe Pm	FAM-5′ TTATTGTCCTCTGGGTTTGGAACTTTGCC 3′- BHQ1
		*P malariae* F	5′ CCAAGCCCAGATAATAAGGAAGGT 3′
		*P malariae* R	5′ TTCGTGCACTTCAACTTACATTCAGT 3′

**Table 3:** Comparison of the Vitassay qPCR Malaria 5 and the homemade real-time PCR results.

Vitassay	Homemade Real-Time PCR					
	*P. falciparum*	*P. vivax*	*P. ovale*	*P. malariae*	Negative	Total
Positive	153 + 1 ^a^	5	20 + 1 ^c^	15	0	195
Negative	1 ^b^	0	0	1 ^c^	3	5
Total	155	5	21	16	3	200

^a^ Isolate identified as *P. ovale* using homemade real-time PCR and detected as *P. falciparum and P. ovale* using the Vitassay qPCR Malaria 5 test. ^b^ Isolate identified *P. falciparum* using homemade real-time PCR and negative using the Vitassay qPCR Malaria 5 test. ^c^ Isolate identified as *P. falciparum and P. malariae* using homemade real-time PCR and detected as *P. falciparum* and *P. ovale* using the Vitassay qPCR Malaria 5 test.

**Table 4:** Sensitivity and specificity of the Vitassay qPCR Malaria 5 for each *Plasmodium* species.

Plasmodium Species	Sensitivity ^a^ Vitassay qPCR Malaria 5	Specificity ^b^ Vitassay qPCR Malaria 5
*P. falciparum*	99.35%	97.72%
*P. vivax*	100%	100%
*P. ovale*	100%	99.44%
*P. malariae*	93.75%	100%

^a^ Sensitivity: TP/(TP + FN). ^b^ Specificity: TN/(FP + TN).

## Data Availability

The datasets analysed in this study are available from the corresponding author upon reasonable request.
